# A Deep Learning-Based Ensemble Method for Early Diagnosis of Alzheimer’s Disease using MRI Images

**DOI:** 10.1007/s12021-023-09646-2

**Published:** 2023-12-02

**Authors:** Sina Fathi, Ali Ahmadi, Afsaneh Dehnad, Mostafa Almasi-Dooghaee, Melika Sadegh, for the Alzheimer’s Disease Neuroimaging Initiative

**Affiliations:** 1 Department of Health Information Management, School of Allied Medical Sciences, Shahid Beheshti University of Medical Sciences, Tehran, Iran; 2 Surrey Business School, University of Surrey, Guildford Surrey, GU2 7XH UK; 3 School of Health Management and Information Sciences, Iran University of Medical Sciences, Tehran, Iran; 4 Neurology Department, Firoozgar Hospital, School of Medicine, Iran University of Medical Sciences, Tehran, Iran

**Keywords:** Alzheimer’s disease, Mild Cognitive Impairment, Deep learning, Convolutional neural networks, Magnetic resonance imaging, Transfer learning

## Abstract

Recently, the early diagnosis of Alzheimer’s disease has gained major attention due to the growing prevalence of the disease and the resulting costs imposed on individuals and society. The main objective of this study was to propose an ensemble method based on deep learning for the early diagnosis of AD using MRI images. The methodology of this study consisted of collecting the dataset, preprocessing, creating the individual and ensemble models, evaluating the models based on ADNI data, and validating the trained model based on the local dataset. The proposed method was an ensemble approach selected through a comparative analysis of various ensemble scenarios. Finally, the six best individual CNN-based classifiers were selected to combine and constitute the ensemble model. The evaluation showed an accuracy rate of 98.57, 96.37, 94.22, 99.83, 93.88, and 93.92 for NC/AD, NC/EMCI, EMCI/LMCI, LMCI/AD, four-way and three-way classification groups, respectively. The validation results on the local dataset revealed an accuracy of 88.46 for three-way classification. Our performance results were higher than most reviewed studies and comparable with others. Although comparative analysis showed superior results of ensemble methods against individual architectures, there were no significant differences among various ensemble approaches. The validation results revealed the low performance of individual models in practice. In contrast, the ensemble method showed promising results. However, further studies on various and larger datasets are required to validate the generalizability of the model.

## Introduction

Dementia is an umbrella term for a group of neurological diseases in which cognitive capabilities deteriorate over time. Alzheimer’s disease (AD), the most common type of dementia, includes 60 to 80 percent of all dementia cases (Jain et al., 2019; Ramzan et al., 2020). It is a progressive and irreversible neurodegenerative disease associated with symptoms such as a decline in cognitive functionality, deficiency of memory, and disturbance of daily activities (Jin et al., 2020). Albeit there is no compelling evidence for the leading cause of AD, it has been reported that some pathophysiological changes in the brain, beginning several years before the final stage, are responsible for the occurrence of AD. These changes comprise the emersion of neurofibrillary tangles within the neurons, which leads to the death of neurons and the accumulation of amyloid plaques among nerve cells, disturbing the usual path of neurotransmitters (Janghel & Rathore, 2021; Liu et al., 2015; Menikdiwela et al., 2018).

Mild Cognitive Impairment (MCI), an intermediate stage between AD and normal control (NC), refers to a detectable decline in cognitive abilities with no disruption in the patient’s daily life (Sarraf et al., 2019). Although not all individuals with MCI progress to AD and dementia, there is a high rate of probability for the conversion from MCI to AD. Therefore, in many studies, MCI has been recognized as the prodromal stage of AD (Abrol et al., 2020; Gorji & Kaabouch, 2019).

Due to the growing demands of global life, the prevalence rate of age-related diseases such as AD has increased in recent years (Lu et al., 2018). The death rate of heart disease and prostate cancer has decreased in the last two decades; meantime, the death rate of AD has increased by 145 percent, making AD the sixth leading cause of death in the US (Association, 2019; Basheer et al., 2021; Nawaz et al., 2021). Although some recent studies have shown promising results for new drugs against AD, there is still no approved treatment for the disease (Hu et al., 2016). As mentioned above, finding a way to diagnose AD accurately in the early stages can have many benefits, including stopping or decreasing the progression of the disease, reducing healthcare costs, and improving people’s quality of life.

To the authors’ best knowledge, there are three different approaches to diagnosing AD. In the first one, which is the most popular due to its ease of use and low cost, specialists utilize clinical information, symptoms, and other criteria like cognitive assessment scales and questionnaires to diagnose AD. However, this approach has some major drawbacks, such as being influenced by subjective factors and having undesirable performance results (Sun et al., 2021). In the second approach, the clinical biomarkers, including the level of tau and amyloid-beta proteins, are measured through the cerebrospinal fluid (CSF) or brain autopsy. Despite its acceptable performance, this approach usually requires invasive procedures for measurement, making it unpopular as a routine method for early diagnosis of AD (Basheera & Ram, 2021; Sun et al., 2021). In the third approach, neuroimaging modalities such as MRI, fMRI, and PET are used to show the structure and functionality of the brain. This method can provide large amounts of information in a short period of time; however, interpreting all the detailed information in images is relatively challenging for physicians (Basheera & Ram, 2021; Sun et al., 2021).

Advances in computing power and the availability of open-access AD-related datasets, have led to the use of machine learning (ML) approaches in the context of early diagnosis of AD (Pellegrini et al., 2018). Deep learning (DL) has recently received much attention due to its brilliant results in different fields and medical image analysis as well (Basaia et al., 2019). DL approaches can extract high-level features, shown to be more efficient than other traditional approaches in a number of studies (Liu et al., 2015). In parallel with the widespread use of DL in various fields, especially medicine, its application in AD diagnosis has recently emerged. In this regard, Suk et al. (Suk & Shen, 2013) conducted the first study on the use of DL in AD diagnosis in 2013. They used the stacked auto-encoder (SAE) method and support vector machine (SVM) classifier in the feature extraction and classification steps, respectively (Suk & Shen, 2013). The following paragraphs summarize some similar studies. You can also see our recent systematic review on the current status of using DL in the early diagnosis of AD for a more comprehensive overview (Fathi et al., 2022).

Li et al. (2021a, b) aimed to diagnose AD through a hippocampal shape and asymmetry analysis by cascaded convolutional neural networks (CNN). Compared to their previous study (Cui & Liu, 2019), which used only hippocampal shape features for classification, their performance was slightly lower this time. Both Mehmood et al. (2021) and Kang et al. (2020) used a 2D-CNN-based architecture called VGG and transfer learning for early diagnosis of AD; however, Kang et al. utilized a multi-modal (MRI/DTI) approach.

ResNet, as the most popular CNN architecture in the literature, was used in a number of studies such as Abrol et al. (2020), Ramzan et al. (2020), Odusami et al. (2021), Shanmugam et al. (2022), Li et al. (2021b), Ji et al. (2019) and Jabason et al. (2019). Some of these studies used the ResNet and other DL methods as an ensemble method (Jabason et al., 2019; Ji et al., 2019) or comparative analysis (L et al., 2023; Li et al., 2021a, b; Odusami et al., 2021; Shanmugam et al., 2022). In the study of Zhang et al. (2021), a 3D-ResNet with the attention mechanism, was proposed to create an explainable model for early AD diagnosis. A few other studies have also utilized the attention mechanism mainly aimed at adding explainability to the black box nature of CNN-based models (Guan et al., 2022; Ji et al., 2020; Liu et al., 2022; Zhang et al., 2021a, b, c, 2022). According to the literature, VGG and DenseNet were the second and third most popular CNN architectures. Most studies, using VGG, employed its standard versions, VGG16 or VGG19; however, some studies proposed customized versions, including the studies of Zhang et al. (2021a, b, c) and Yu et al. (Yu et al., 2019).

DenseNet, one of the most successful architectures for early AD diagnosis, was used in some recent studies. Li and Liu (2018, 2019) and Liu et al. (2020) applied 3D-DenseNet in their studies in order to extract high-level features and classify different stages of AD. The authors in Li and Liu (2018) extracted features from various parts of the brain using patch-based strategies, whereas in Li and Liu (2019) and Liu et al. (2020), only the hippocampus region was used to extract features. Several DenseNet architectures are combined in ensemble approaches reported in the studies of Wang et al. (2019), Ruiz et al. (2020), and Islam and Zhang (2018). Some reviewed studies have proposed customized CNN architectures to diagnose AD and its prodromal stages, MCI or its subcategories, namely the early MCI (EMCI) and late MCI (LMCI). Basaia et al. (2019) have proposed a 3D-CNN with 12 convolutional blocks, a rectified linear unit (ReLu) as the activation layer, a fully connected layer, and a logistic regression layer as the classifier for automatic classification of AD and subcategories of MCI. Gorji and Kaabouch (2019) developed a simple 2D-CNN architecture with three convolution layers, each followed by a max-pooling, a fully-connected layer, and a sigmoid classifier for binary classification of prodromal stages of AD. The study focused on gray matter (GM) due to its proven effect on the early onset of AD. Pan et al. (2020) employed an ensemble scheme based on different 2D-CNN classifiers for early detection of AD.They built various base CNN classifiers on single-axis slices of MR images and created an ensemble model based on the five best classifiers for each axis.

In general, the findings in the literature are promising; several studies have demonstrated high accuracy in classifying normal controls, patients with AD, and patients with MCI using deep learning models. This suggests that deep learning can be a valuable tool for early detection of AD. Hence, the motivation for the current study is two-fold. First, early diagnosis of Alzheimer’s disease is critical for improving patient outcomes. The earlier the disease is diagnosed, the sooner the treatment can be initiated. This can help slow the progression of the disease and improve quality of life for patients and their families. Second, early diagnosis of Alzheimer’s disease can help to reduce healthcare costs.

While many reviewed studies reported promising results, most did not address all clinically valuable classification groups. In addition, there is no comprehensive comparative analysis among various individual base classifiers and ensemble approaches. In the current study, we aimed to propose an ensemble method based on deep learning for the early diagnosis of AD using MRI images. The proposed method was comprised of six well-known convolutional neural networks (CNN) based on a novel approach called the weighted probability-based ensemble method (WPBEM). The main contributions of the current study are described as follows:

1. A novel ensemble method called WPBEM was used to enhance the performance of the individual CNN models for early diagnosis of AD.
2. A comparative analysis was utilized to find hyperparameters and the optimal scenario for combining the individual CNNs.
3. In order to enhance the performance of the models, we used a domain adaptation transfer learning approach producing superior results to any other parameter initialization methods.
4. The current study has addressed all the valuable binary and multiclass classification groups.

According to the aforementioned reasons, the main aim of the current study was to propose an ensemble method called WPBEM based on different base CNN architectures for the early diagnosis of AD. This is a novel approach for two reasons. First, many of the previous studies used individual CNN architectures or ensemble methods with a single type or less than three types of base CNN classifiers. However, the current study used six different types of CNN classifiers. The number and types of base classifiers were selected through a comparative analysis of well-known CNN architectures. Secondly, instead of using simple majority voting or bagging in the ensemble method, we used a weight variable for the outputs of each model, presenting the correctness of each disease class. We also used a local dataset for validating the created models.

## Methodology

### Collecting Data and Preprocessing

In this study, we employed an end-to-end deep learning-based scheme comprised of different predefined and modified 2D-CNN architectures called WPBEM for early AD diagnosis. Two independent datasets were used in this study. The first one was gathered from Alzheimer’s Disease Neuroimaging Initiative (ADNI) dataset (adni.loni.usc.edu) for training and evaluation of models, and the second one was collected from Firoozgar hospital in Tehran, Iran and was used for validation of the model previously created by ADNI dataset. The ADNI was launched in 2003 as a public–private partnership, led by Principal Investigator Michael W. Weiner, MD. The primary goal of ADNI has been to test whether serial magnetic resonance imaging (MRI), positron emission tomography (PET), other biological markers, and clinical and neuropsychological assessment can be combined to measure the progression of mild cognitive impairment (MCI) and early Alzheimer’s disease (AD). For up -to-date information, see www.adni-info.org. The data were T2-weighted MRI images, taken in axial view and comprised 721 subjects from ADNI and 26 from the local dataset (Firoozgar hospital). There were five groups of subjects in the ADNI data, namely NC, MCI, EMCI, LMCI, and AD, in which the MCI group was not used for binary classification but only for three-way classification. Meanwhile, the local dataset consisted of three classes: NC, MCI, and AD. The demographic details of the participants are shown in Table 1.

**Table 1:** The demographic details of participants and the number of slices extracted from each image

Dataset	Diagnostic type	Number	Gender (M/F)	Age (Avg)	Number of Slices
ADNI	NC	132	72/60	77.18	2610
	MCI	157	79/78	75.92	3130
	EMCI	135	70/65	73.33	2625
	LMCI	131	69/62	74.09	2565
	AD	166	81/85	75.87	3311
Local dataset	NC	4	1/3	65.37	36
	MCI	11	4/7	71.36	101
	AD	11	4/7	84.82	101

After collecting the images from mentioned datasets, the preprocessing steps, including normalization, resizing, removing non-brain slices, selecting slices with the most information, and converting 3D images into 2D slices, were conducted on collected images. During preprocessing, the intensities of each slice were rescaled to 0–1 by Eq. (1) to achieve intensity normalization. Since most slices had a dimension of 256 × 256 by default, all slices were resized to this dimension to preserve maximum information during feature extraction.

1 $$\widehat{X}= \frac{(X-{X}_{min})}{{X}_{max}-{X}_{min}}$$

**Fig. 1:**
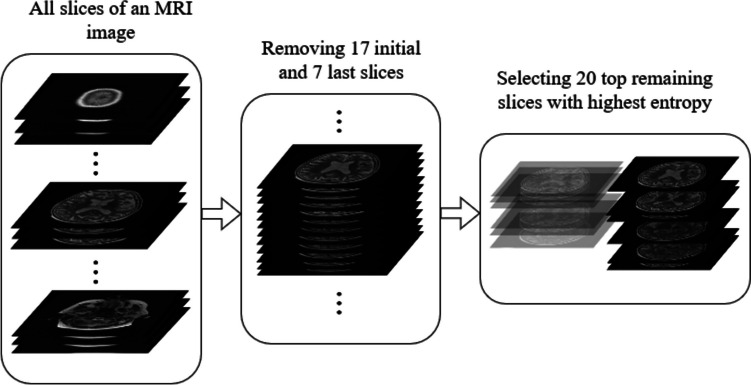
The process of selecting the most informative slices from the raw MRI image of each subject for creating the final dataset

Next, some of the first and last slices of each 3D image were ignored by consulting a neurologist if they were not valuable for AD detection. For example, in the case of images in the ADNI dataset, 17 initial and seven last slices were removed. Among the remaining volume, 20 slices with highest entropy, the most informative slices, were selected for the final dataset. The procedure for selecting slices for the final dataset is shown in Fig. 1. All the preprocessing steps were written in Python 3.7 and some of its libraries, including Numpy, Pydicom, Globe, Opencv, and Scikit-image.

### The Proposed Model

After preprocessing the images and providing the final dataset, a deep learning-based ensemble approach was employed for the early detection of AD. Slice-based strategy, the most popular feature extraction strategy in the literature, was used in this study due to its straightforwardness, low complexity, and no need for complex preprocessing, which is more consistent with the end-to-end nature of our approach. Moreover, converting 3D images into 2D slices led to a dataset with more samples, which is essential for deep learning to prevent overfitting and maintain the generalizability of models.

As mentioned before, the proposed method was based on CNN architectures. Given that combining multiple classifiers offers superior results for AD detection, six different base classifiers were combined in this study. These classifiers were inspired by well-known CNN architectures, namely DenseNet201, DenseNet169, DenseNet121, ResNet50, Inception-Resnet V2, and VGG19, chosen because of their promising results in the previous studies reviewed by authors. By modifying the latest layers of architecture, each was adapted to the current research situation.

**Fig. 2:**
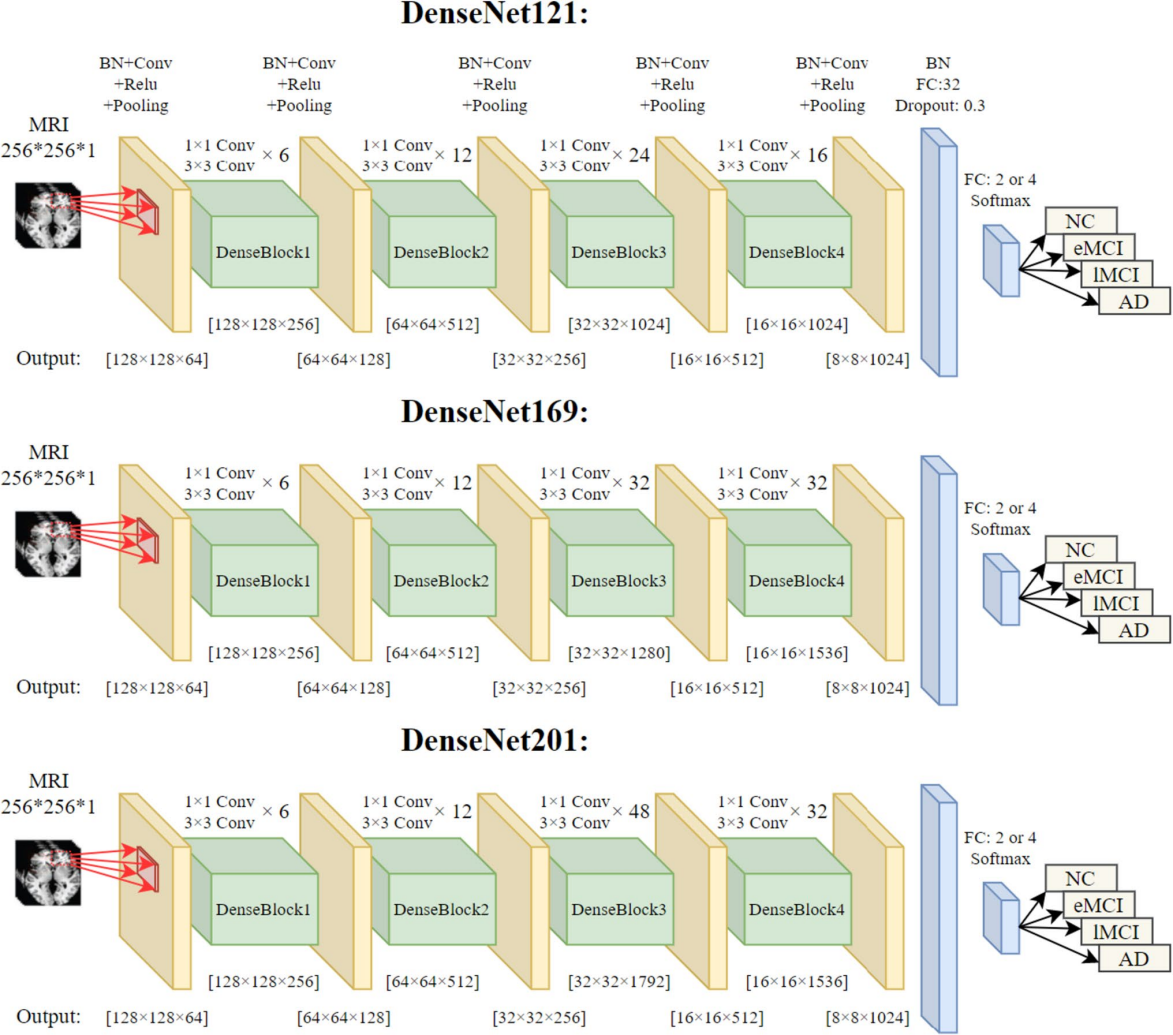
The structure of the proposed DenseNet architectures

DenseNets utilize a base structure named dense block in which each preceding layer of this block is connected simply to all the next layers. This structure improves information flow throughout the network and solves the gradient vanishing problem. The standard version of DenseNet architectures consisted of four dense blocks, five transform layers, one fully connected layer, and one softmax layer as a classifier. In different versions of predefined DenseNets, such as DenseNet201, DenseNet,169, etc., the structure and number of inner layers of dense blocks are different. You can see our proposed DenseNet architectures in Fig. 2.

As shown in Fig. 2, we have replaced the last layer of the standard version of DenseNets with a batch normalization layer followed by a fully connected layer with 32 neurons, a dropout layer with a value of 0.3 and a softmax layer.

**Fig. 3:**
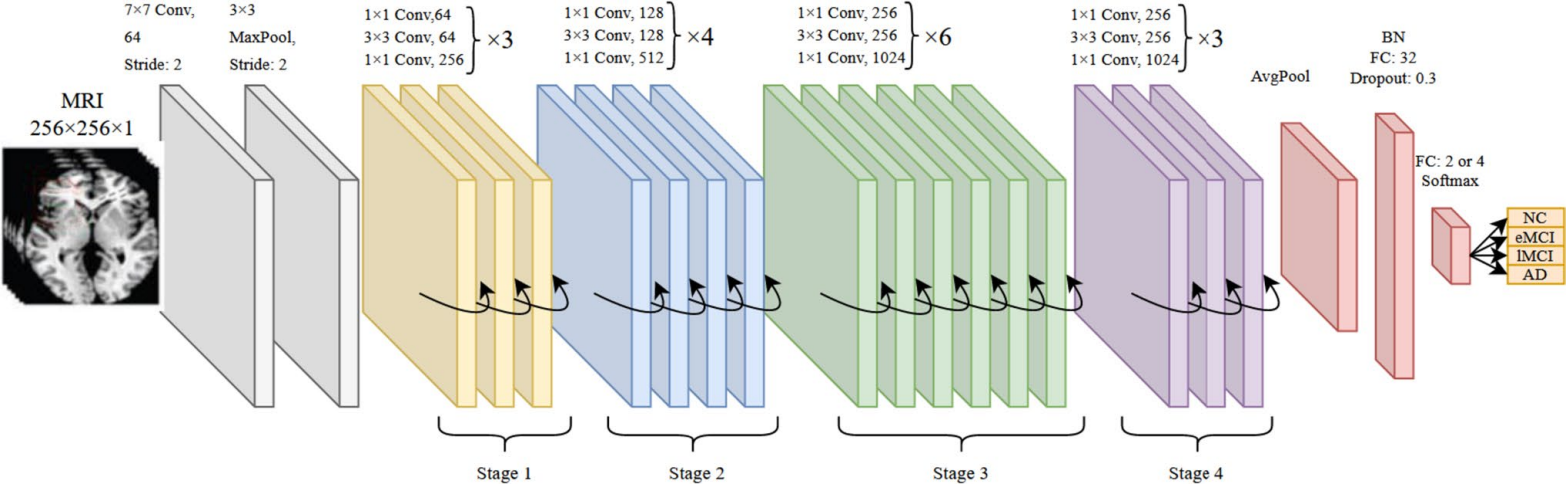
The structure of the proposed ResNet architecture

Similar to DenseNet, ResNet was also employed to speed up the convergence of the model and address the vanishing gradient problem. This is done by making shortcut connections between layers. Our modified ResNet architecture was inspired by ResNet50 and consisted of four stages with three, four, six, and three residual blocks, followed by layers similar to DenseNets’ latest layers added at the end (see Fig. 3).

**Fig. 4:**
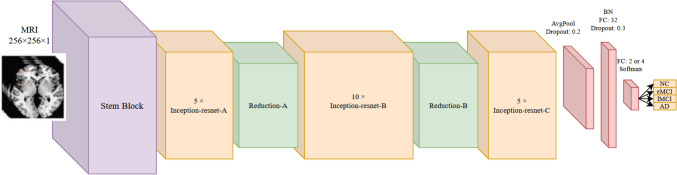
The structure of the proposed Inception-ResNet architecture

As shown in Fig. 3, the structure of residual blocks in various stages is similar in relation to the number of layers but different with regard to the number of kernels. Just like the previously proposed architectures, we have modified the standard version of Inception-ResNet V2 as another individual architecture participating in the ensemble model. You can see the simplified structure of Inception-ResNet in Fig. 4.

**Fig. 5:**
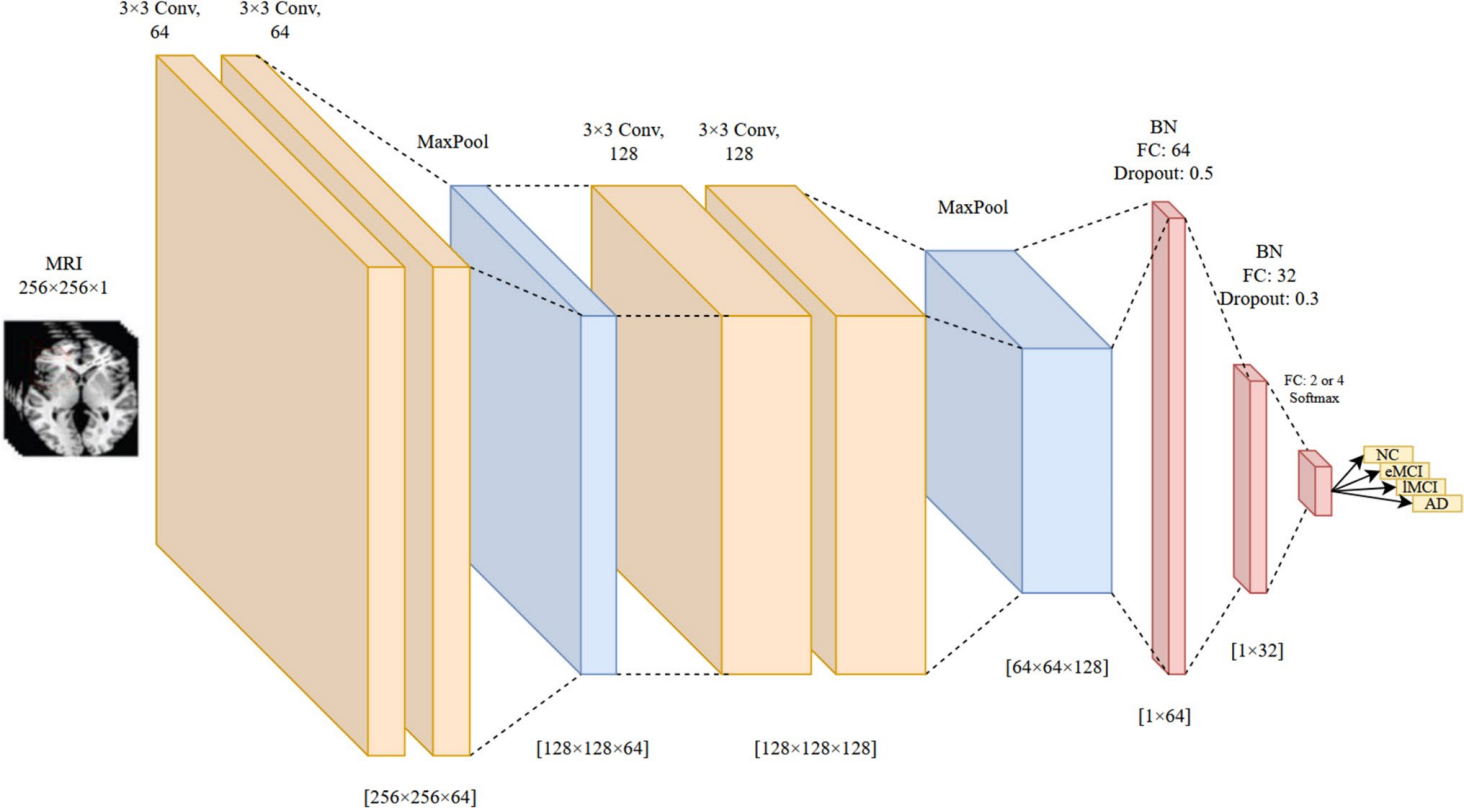
The structure of the proposed VGG architecture

The last individual architecture in the proposed ensemble model was VGG19. As shown in Fig. 5, only six initial layers of the standard version are used in the modified architecture due to speeding up the converging time, reducing the number of parameters, computational cost, and probability of overfitting. Additionally, we added two batch normalization layers, two fully connected layers, two dropout layers, and one softmax layer to the model.

### Ensemble Learning

**Fig. 6:**
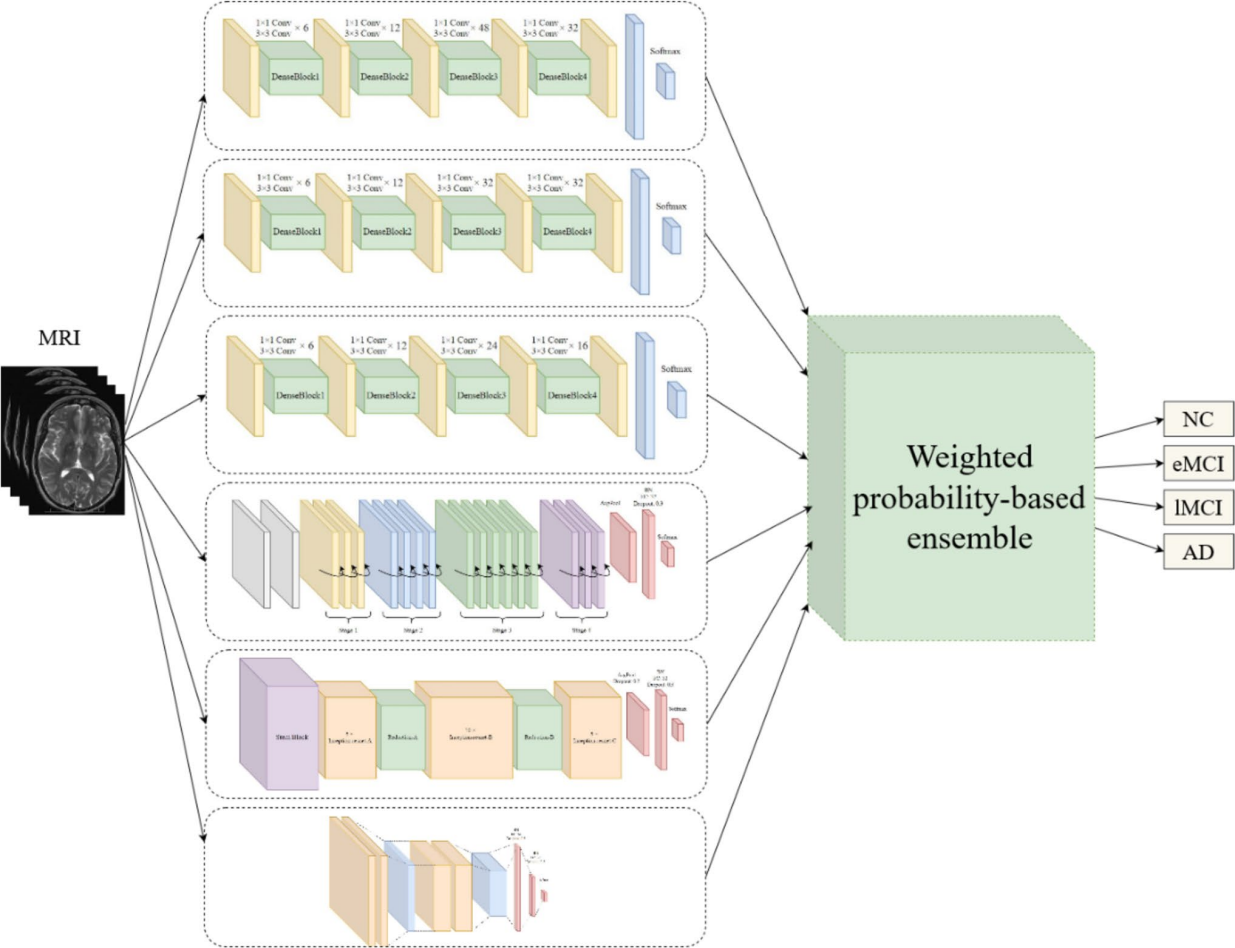
The scheme of the proposed WPBEM model

The proposed ensemble method consisted of two phases. In the first one, all base classifiers mentioned above were individually trained and evaluated on the same training and test datasets. Then in the second phase, the weighted probability-based ensemble method was utilized to combine the base classifiers. The overall scheme of the proposed model is shown in Fig. 6.

The accuracy of each classifier in the first phase was used as the weight of that classifier in the final model; in other words, the more accurate the classifier, the greater its effect in the final ensemble model. Next, the probabilistic value of each class in individual classifiers was multiplied by the weight value of the related classifier. The model output was obtained by applying a final softmax function to the sum of weighted probabilities. Hence, the output was the class with the highest probability in the final softmax function. The procedures are defined as follows:

2 $${O}_{j}=\textstyle\sum_{i=1}^{6}{w}_{i}\times {\alpha }_{j}^{i} \quad where \quad j=1,\dots ,4 \quad and \quad i=1,\dots ,6$$

where *i* is the index of each classifier, *j* is the index of each class, ${w}_{i}$ indicates the weight (accuracy) of *i*th classifier, ${\alpha }_{j}^{i}$ indicates the probability value of *j*th class in the *i*th classifier, and ${O}_{j}$ is the sum of weighted probabilities for *j*th class. The model output obtains from:

3 $$P=softmax \left({O}_{j}\right) \quad where \quad j=1,\dots ,4$$

4 $$R=argMax \left({P}_{j}\right) \quad where \quad j=1,\dots ,4$$

where *P* is the output of the softmax function, and *R* is the final output of the ensemble method.

### Transfer Learning and Fine-Tuning

In order to enhance the performance of the proposed model and speed up the training time, a domain adaption-based transfer learning methofd and fine-tuning were used in this study. In the domain adaption approach, although the source (initial) and target (original) datasets are different, they are in the same domain. Hence, in this study, firstly, the deep model was trained by NC/AD binary classification group with a random initialization method, then the parameters of this trained model were used for other classification groups. Transferring and fine-tuning parameters were deployed in two stages described below:

In transferring phase, the initial convolutional blocks and layers of the pre-trained model (NC/AD classification model) for each of the individual classifiers were frozen (got untrainable), and only the latest custom layers remained trainable. The model was re-trained by the new classification group with a learning rate of 0.001. So the transferred version of the model was obtained. In the second stage, called tuning, all layers and convolutional blocks of the transferred version were unfrozen, the learning rate decreased to 0.0001, and the model was re-trained again to obtain the final fine-tuned version of model.

### Evaluation

After the training step, the models were evaluated by performance metrics, namely accuracy, sensitivity, and specificity, the calculation of which is given in Eqs. (5)–(7). A split ratio of 80:20 was used for the training and test sets in this study, with 10% of the training set being utilized as validation.

5 $$Accuracy=(\mathrm{TP}+\mathrm{TN}) /(TP+TN+FN+FP )$$

6 $$Sensitivity=\mathrm{ TP}/(TP+FN)$$

7 $$Specificity=\mathrm{ TN}/TN+FP$$

where true positive (TP) is the number of patients diagnosed correctly by the deep model, also, true negative (TN) indicates how many non-patients were correctly diagnosed, false positive (FP) indicates how many non-patients were misdiagnosed, and false negative (FN) implies how many patients were misdiagnosed. Besides the performance metrics mentioned above, we used the receiver operating characteristic (ROC) curve to compare the performance of the proposed ensemble model with individual models.

## Experimental Results

The performance of ensemble model and its constituent models was evaluated and compared on various binary and multiclass classification groups, including NC/AD, NC/EMCI, EMCI/LMCI, LMCI/AD, 4-way (NC/EMCI/LMCI/AD), and 3-way classification groups (NC/MCI/AD).

### Selecting Hyperparameters

By conducting exploratory analysis and reviewing previous literature, we were able to select appropriate hyperparameter values. In Table 2, some of the selected hyperparameters are shown.

**Table 2:** Selected values for some of the hyperparameters

**Hyperparameter**	**Value**
Optimization algorithm	Adam
Loss function	Cross-entropy
Learning rate	0.001, 0.0001
Batch size	32, 64
The number of epochs	50,100,200,300,350

**Fig. 7:**
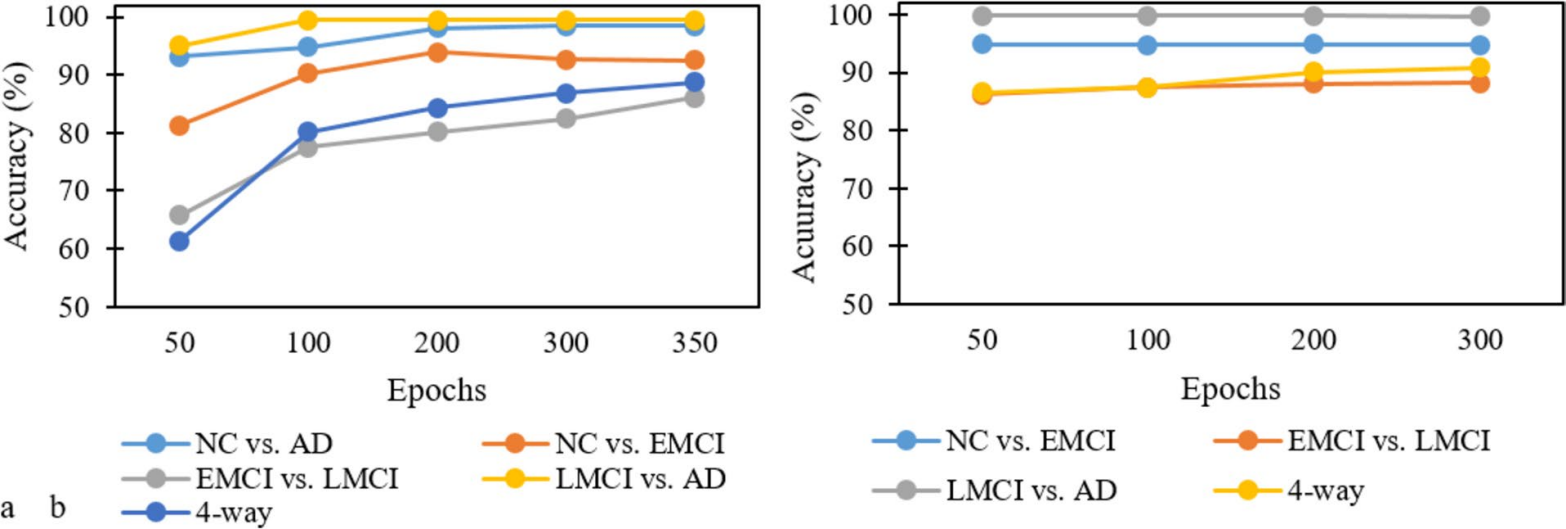
Explorative analysis of the number of epochs based on DenseNet121 architecture – **a** The accuracy obtained for different numbers of epochs in random parameters initialization method – **b** The accuracy obtained for different numbers of epochs in fine-tuned models

Although various optimization algorithms such as stochastic gradient descent (SGD), Adam, Adadelta, Adagrad, and root mean squared propagation (RMSProp) have been used in deep models, Adam and SGD are the most popular algorithms in the literature (Ebrahimighahnavieh et al., 2020). In this study, we utilized Adam due to its lower computational cost than other algorithms. The most popular loss functions used in the reviewed studies were mean square error (MSE) and cross-entropy. However, in classification models with a softmax output layer, cross-entropy has been found to perform better than MSE (Sadowski, 2016). The default learning rate value of Keras framework (0.001) was used in the initial training phase and then decayed to 0.0001 for fine-tuning the parameters. For batch size, we chose 64 as an initial value to speed up training time, but it was reduced to 32 when encountering an out-of-memory (OOM) error. The number of epochs was chosen exploratively according to the challenging level of the classification groups in the initial training phase. Thus, more epochs were required when the classification group was more challenging. For example, the model usually converged to an optimal solution in much less time in fine-tuning than in initial training; therefore, fewer epochs were needed. You can see the explorative investigation into the required number of epochs for model training in Fig. 7. We utilized the DenseNet-121 as the base architecture for the exploratory investigation. The proper values obtained were then generalized to other architectures.

As shown in Fig. 7, the random strategy for parameter initialization requires more time to converge (200 to 350 epochs) compared to fine-tuned models that converges in less than 100 epochs. Regarding the classification groups, as it can be observed, some of the classification groups, such as EMCI/LMCI and 4-way classification, converge later than others because of their challenging nature, thus requiring more epochs.

**Fig. 8:**
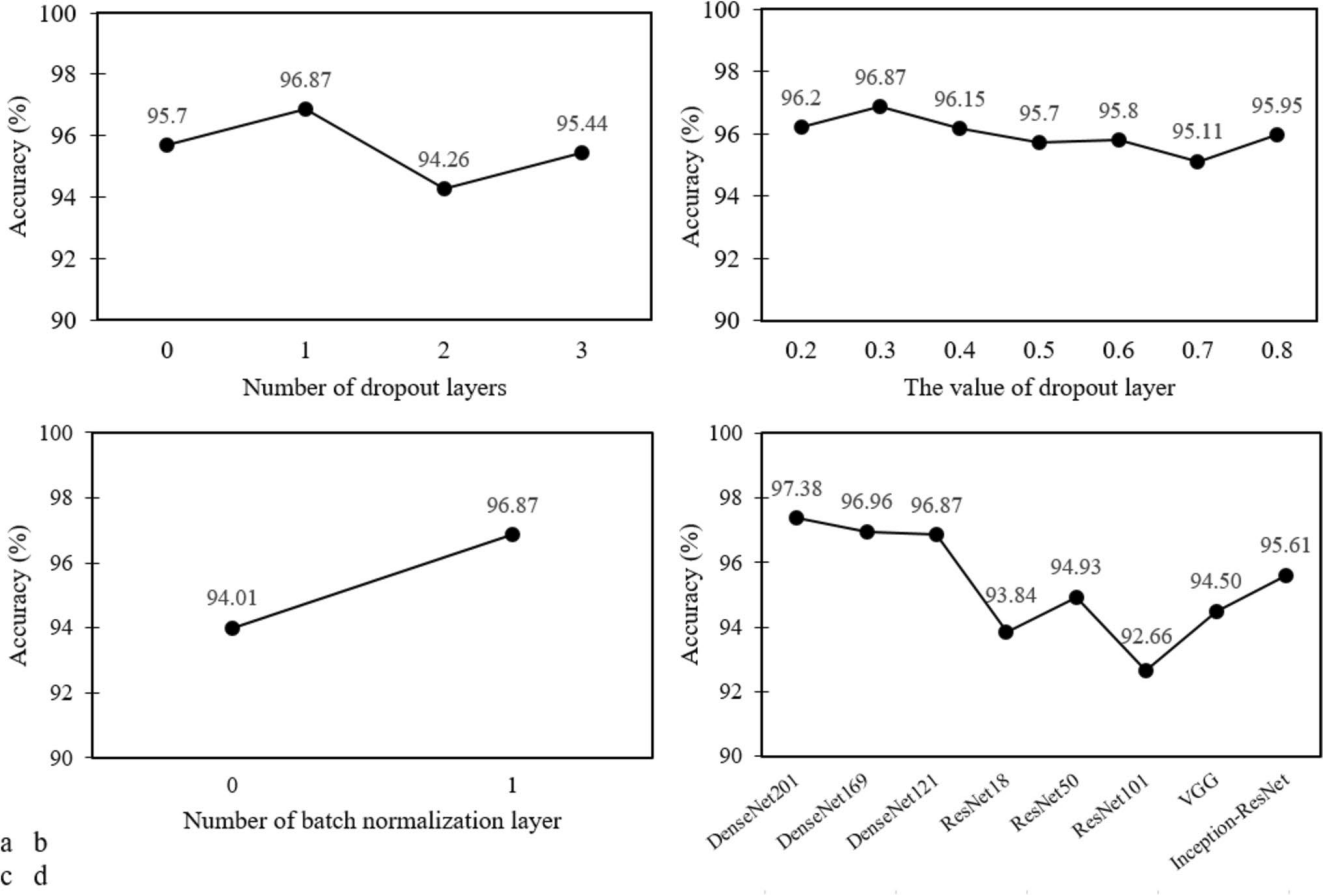
Explorative analysis on hyperparameters – **a** Checking the model accuracy in using different numbers of dropout layers – **b** Checking the model accuracy in using different values for dropout layer – **c** The effect of using batch normalization layer on model accuracy – **d** Comparing the accuracy of various well-known CNN architectures

We performed more deep explorative analysis for selecting other hyperparameters, such as the number and value of dropout layers, the number of batch normalization layers, and selecting the type and number of base classifiers for the ensemble model. To this end, we used NC/AD classification group and DenseNet121 as the base group and classifier to check the different states of hyperparameters (See Fig. 8).

**Fig. 9:**
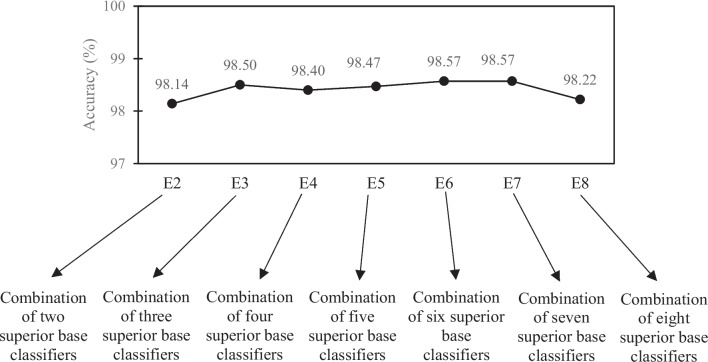
The assessment of different scenarios of the ensemble model

As shown in Fig. 8, the model with one dropout layer, a value of 0.3, and one batch normalization layer performs better than other states. We also compared popular CNN architectures, and as shown in Fig. 8d, the DenseNet-based models outperformed other architectures. The following scenarios for combining individual CNN classifiers, according to results shown in Fig. 8d, were defined and assessed to obtain the best ensemble model. You can see the scenarios and their performances in Fig. 9.

As shown in Fig. 9, the fifth and sixth scenarios (E6 and E7) has the highest performance (acc = 98.57) among the seven defined scenarios. Hence, we chose the E6 scenario (ensemble of DenseNet201, DenseNet169, DenseNet121, ResNet50, VGG, and Inception-ResNet) as the final ensemble model due to its lower computational cost against E7.

### Performance Metrics

After selecting the proper hyperparameters, the base classifiers and ensemble method were trained and evaluated. In order to address the early diagnosis of AD, we developed various binary and multiclass classification groups: Table 3 reports the binary and four-way classification performance of models.

**Table 3:** Binary and four-way classification performance on the test set (ADNI)

**Method**	**NC vs. AD**			**NC vs. EMCI**			**EMCI vs. LMCI**			**LMCI vs. AD**			**4-way**
	**Acc**	**Sen**	**Spe**	**Acc**	**Sen**	**Spe**	**Acc**	**Sen**	**Spe**	**Acc**	**Sen**	**Spe**	**Acc**
DenseNet201	97.38	96.59	98.43	94.94	96.26	93.55	88.15	86.83	89.49	99.83	**99.79**	100	90.87
DenseNet169	96.96	97.19	96.67	95.7	98.32	92.97	90.75	89.89	91.63	99.74	99.55	100	89.29
DenseNet121	96.88	97.78	95.68	96.08	98.13	93.95	89.79	88.74	90.56	99.57	99.7	99.4	88.48
ResNet50	94.94	94.67	95.29	94.08	97.94	90.04	84.2	80.34	88.13	98.98	98.96	99	86.55
VGG	94.51	93.19	96.27	94.94	99.07	90.63	88.15	89.89	86.38	98.98	98.22	100	87.99
Inception-ResNet	95.61	95.11	96.27	94.17	96.63	91.60	84.68	84.16	85.21	98.89	98.96	98.8	88.80
MVEM	98.31	97.63	99.22	96.37	99.63	92.97	93.74	91.6	**95.91**	99.66	99.4	100	**93.93**
PBEM	98.5	98.72	**98.35**	96.37	99.63	92.97	94.03	93.7	94.36	99.83	99.7	100	93.83
**WPBEM**	**98.57**	**98.81**	98.24	**96.37**	**99.63**	**92.97**	**94.22**	**94.08**	94.36	**99.83**	99.7	**100**	93.88

Bold values represent the maximum value in each column

*MVEM* majority-voting ensemble method, *PBEM* probability-based ensemble method, *WPBEM* weighted probability-based ensemble method (Proposed method)

**Fig. 10:**
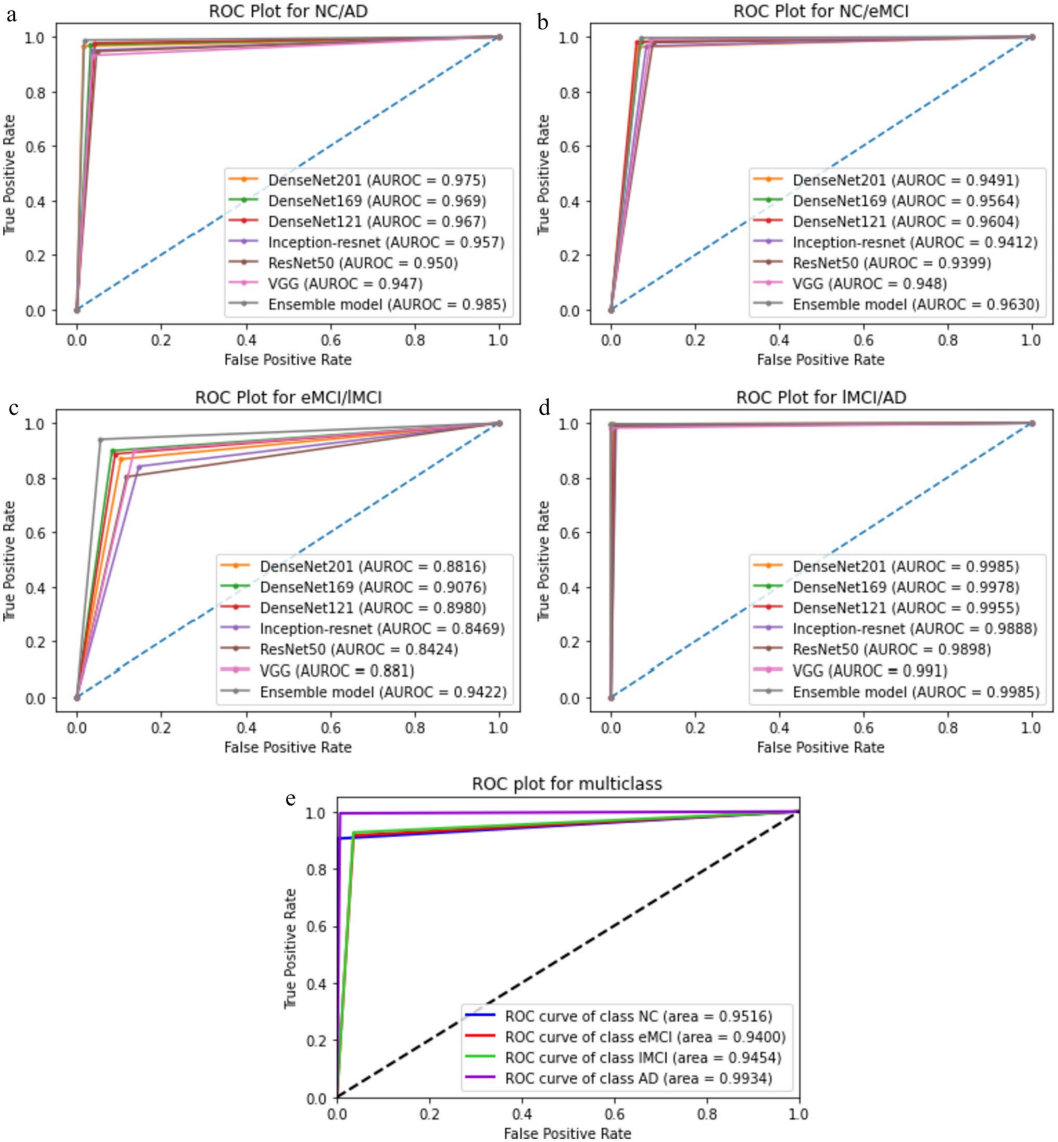
ROC plot for **a** NC/AD classification group – **b** NC/EMCI classification group – **c** EMCI/LMCI classification group – **d** LMCI/AD classification group – **e** Four-way classification group

Based on Table 3, the performance results of all ensemble methods (the last three rows) were superior to individual models. Comparative analysis of ensemble approaches revealed no significant differences in classification performance; however, the proposed model (WPBEM) performed slightly better than other common ensemble approaches in most classification groups. Figure 10 shows the ROC plot for all the individual models and the proposed ensemble model in every classification group.

As shown in Fig. 10, the most challenging group for classification is EMCI/LMCI, in which the ensemble model significantly outperforms the individual classifiers. Note that the ROC curve cannot be drawn directly for multiclass classification models; therefore, in the 4-way classification model, ROC curves were drawn individually for each class. The proposed ensemble method demonstrated acceptable performance in detecting all disease classes in the 4-way classification group; however, it proved to be more appropriate for detecting AD and NC (See Fig. 10e).

### Validating on Local Dataset

**Fig. 11:**
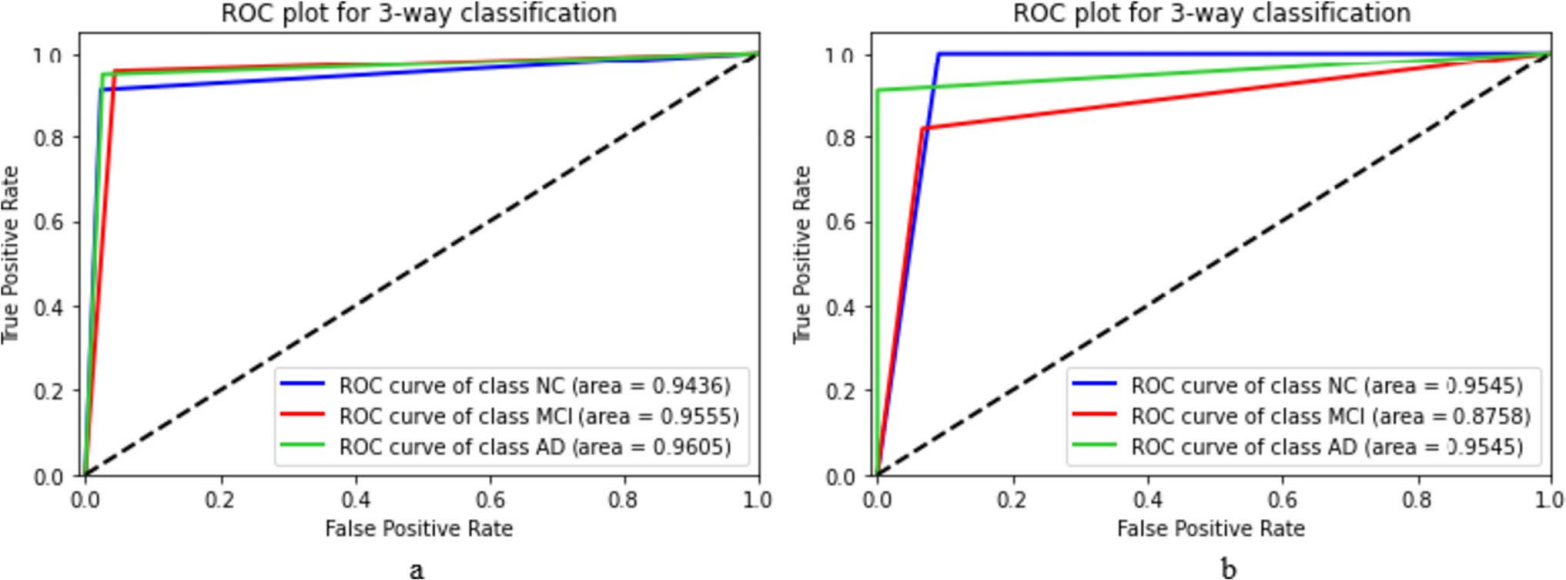
ROC plot for three-way classification – **a** Evaluating the model based on ADNI data – **b** Validating the model based on local dataset

The local dataset consisted of three disease classes (NC, MCI, AD), so we first trained and evaluated a three-way classification model based on ADNI data and then validated it using the local dataset. Table 4 and Fig. 11 report the performance results for evaluating and validating the propsed model based on ADNI and local datasets.

**Table 4:** Performance results of evaluating and validating the model

	DenseNet201	DenseNet169	DenseNet121	ResNet50	VGG	Inception-ResNet	**WPBEM**
Evaluation^a^	93.08	92.89	92.78	92.05	90.34	87.85	**93.92**
Validation^b^	73.08	69.23	76.92	65.38	69.23	61.54	**88.46**

^a^Accuracy of the model based on ADNI dataset

^b^Accuracy of the model based on local dataset

Although validation results on the local dataset in individual models were not promising, the ensemble model (WPBEM) could enhance the accuracy by at least 15 percent. Even though WPBEM improved the validation results on the local dataset, the accuracy was nearly five percent lower than the evaluation results on ADNI, suggesting that models should be considered further for generalizability. Based on Fig. 10, the WPBEM has performed almost similarly in detecting NC and AD cases in the local and ADNI datasets, but MCI cases in the local dataset appeared more challenging for the model to detect.

## Discussion

This study sought to introduce an ensemble model using deep learning for early diagnosis of AD. It comprised of collecting the dataset, preprocessing, creating the individual and ensemble models, evaluating the models based on ADNI data, and validating the trained model based on the local dataset. The proposed method is a novel ensemble approach selected through a comparative analysis of various ensemble scenarios. Finally, the six best individual CNN-based classifiers were selected to combine and constitute the ensemble model. As mentioned before, the performance metrics used in the current study were accuracy, sensitivity, and specificity. Therefore, these metrics were used to compare the model with state-of-art works. Table 4 summarizes the comparative analysis of the proposed model and some similar studies according to the evaluation of ADNI data.

All valuable classification groups, including binary and multiclass classifications, were addressed in this study. Only two binary classification groups, NC/LMCI and EMCI/AD, were not addressed because they are less clinically valuable than other groups for early AD diagnosis. We only included studies in Table 5 using MRI images as input data and addressed subcategories of MCI (EMCI and LMCI) to compare with our study. None of the reviewed studies addressed all classification groups. Although Mehmood et al. (2021), Basaia et al. (2019), and Yue et al. (2019) have reported all the binary classification groups, they have not addressed the multiclass classification in their studies which is one of the most important classification groups.

**Table 5:** Comparing our proposed model with similar studies

**Study**	**Method**	**NC vs. AD**			**NC vs. EMCI**			**EMCI vs. LMCI**			**LMCI vs. AD**			**4-way**
		**Acc**	**Sen**	**Spe**	**Acc**	**Sen**	**Spe**	**Acc**	**Sen**	**Spe**	**Acc**	**Sen**	**Spe**	**Acc**
Li et al. (2021a, b)	2D-CNN+3D-CNN	85.9	81.5	89.9	-	-	-	71	59.8	79	-	-	-	-
Mehmood et al. (2021)	2D-CNN (VGG)	98.73	98.19	99.09	87.06	86.61	86.63	81.06	80.61	81.52	82.07	81.39	82.4	-
Zhang et al. (2021a, b, c)	3D-CNN (ResAttNet)	91.3	91	91.9	-	-	-	82.1	81.2	80.9	-	-	-	-
Ruiz et al. (2020)	Ensemble of 3D-DenseNets	-	-	-	-	-	-	-	-	-	-	-	-	83.33
Kang et al. (2020)	2D-CNN (VGG)	-	-	-	94.2	97.3	92.9	-	-	-	-	-	-	-
Pan et al. (2020)	2D-CNN	84	-	-	-	-	-	62	-	-	-	-	-	-
Abrol et al. (2020)	3D-CNN (ResNet)	91	-	-	-	-	-	77.8	-	-	-	-	-	-
Basaia et al. (2019)	3D-CNN	98.2	98.1	98.3	76.4	75.1	77.8	74.9	74.8	74.1	75.8	74.8	77.1	-
Gorji and Kaabouch (2019)	2D-CNN	-	-	-	93.96	90.46	98.11	93	91.48	94.82	-	-	-	-
Yue et al. (2019)	2D-CNN	98.6	97.2	-	99.7	99.5	-	98	97.2	-	99.7	99.7	-	-
Lu et al. (2018)	SAE+DNN	84.6	80.2	91.8	-	-	-	82.93	79.69	83.84	-	-	-	-
Liu et al. (2018)	2D-CNN+3D-CNN	93.26	92.55	93.94	64.04	63.07	67.31	-	-	-	-	-	-	-
Islam and Zhang (2018)	Ensemble of 2D-DenseNets	-	-	-	-	-	-	-	-	-	-	-	-	93.18
Shi et al. (2018)	Stacked DPN	97.13	95.93	98.53	-	-	-	78.88	68.04	86.81	-	-	-	57
Suk et al. (2017)	2D-CNN	91.02	92.72	89.94	-	-	-	74.82	70.93	82.78	-	-	-	-
Ortiz et al. (2016)	DBN	90	86	94	80	60	90	-	-	-	-	-	-	-
**Ours**	**Ensemble of six 2D-CNNs**	98.57	98.81	98.24	96.37	99.63	92.97	94.22	94.08	94.36	99.83	99.70	100	93.88

*DPN* deep polynomial network, *DBN* deep belief network

As shown in Table 5, our performance results are higher than most of the reviewed papers and comparable with others. Especially in the four-way classification, the proposed model outperformed other studies. Although Yue et al. reported brilliant performance results in most binary classification groups, their study did not address the multiclass classification.

Regarding the utilized ensemble approach, majority-voting (MVEM) and probability-based ensemble methods (PBEM) have been used in reviewed studies, in which MVEM is more common in the literature (Islam & Zhang, 2018; Jabason et al., 2019; Lu et al., 2018; Sarraf et al., 2019; Zheng et al., 2018). The use of PBEM has also been reported in the studies of Ruiz et al. (2020) and Wang et al. (2019). Using PBEM as an inspiration, a new method called WPBEM has been introduced and compared to other approaches in the current study. Although comparative analysis shows superior results of ensemble methods against individual architectures, which is in line with other studies, there are no significant differences among various ensemble approaches (Mujahid et al., 2023). WPBEM has performed equally well or slightly better in most binary classification groups than other approaches, but MVEM has performed slightly better in four-way classifications (See Table 3). A recent study by Ma et al. (2023) has introduced a novel ensemble approach known as deep-broad ensemble. This method combines 3D-residual convolutional blocks with a broad learning system, demonstrating superior performance compared to individual methods. A significant benefit of the proposed approach is the elimination of the requirement for expensive hardware resources and extended training durations.

## Conclusion and Future Work

With the growing elderly population in recent decades and its consequences, such as increasing age-related diseases, including Alzheimer’s, researchers are concerned about improving and developing new ways to diagnose these diseases at an early stage. Machine learning and deep learning approaches have revealed great potential in diagnosing such diseases. Although deep learning methods, including the proposed model, have achieved competitive performance in diagnostic results, important issues still need to be addressed.

Most studies have deployed and evaluated their DL frameworks based on standard datasets such as ADNI and OASIS, which are beneficial in terms of research value, but using these frameworks practically as a computer-aided diagnosis (CAD) system, requires more investigations. To address this issue, we performed a validation procedure by measuring the accuracy of trained models on a local dataset. The validation results have revealed that individual models show low performance in practice. In contrast, the proposed ensemble method shows promising diagnostic results. However, due to the accuracy difference between ADNI evaluation and local data validation, this model should be used cautiously in practice. Hence, further experiments on various and larger datasets are required to validate the generalizability of the model.

This study focused on introducing a DL framework based on MRI images. However, it could be re-designed and re-trained on the basis of other neuroimaging data, including PET and fMRI. Also, further experiments on developing multi-modal and modality-independent frameworks could be taken into consideration. It is also necessary to investigate the effectiveness of other deep learning methods, such as recently introduced CNN architectures, in the early AD diagnosis.

## Data Availability

The datasets used in this study were achieved from Alzheimer’s Disease Neuroimaging Initiative (ADNI) and Firoozgar hospital in Tehran. You can visit the following link for more information about ADNI: http://adni.loni.usc.edu/.
